# Multidisciplinary programme for rehabilitation of chronic low back pain – factors predicting successful return to work

**DOI:** 10.1186/s12891-021-04122-x

**Published:** 2021-03-06

**Authors:** Gabriel Ching Ngai Leung, Prudence Wing Hang Cheung, Gareth Lau, Sin Ting Lau, Keith Dip Kei Luk, Yat Wa Wong, Kenneth Man Chee Cheung, Paul Aarne Koljonen, Jason Pui Yin Cheung

**Affiliations:** Department of Orthopaedics and Traumatology, The University of Hong Kong, 5th Floor, Professorial Block, Queen Mary Hospital, Pokfulam, Hong Kong SAR, China

**Keywords:** Low Back pain, Return to work, Conservative treatment, Logistic models, Spine

## Abstract

**Background:**

There are no clear indicators for predicting return to work for patients with chronic low back pain (LBP). We aim to report the outcomes of a 14-week multidisciplinary programme targeting patients with chronic LBP who failed conventional physiotherapy to provide functional rehabilitation. Also, this study will identify factors predicting successful return to work (RTW).

**Methods:**

A collected cohort of patients with chronic LBP was consecutively enrolled into the programme from 1996 to 2014. All recruited patients failed to RTW despite at least 3 months of conservative treatment. Patient underwent weekly multidisciplinary sessions with physiotherapists, occupational therapists and clinical psychologists. Patient perceived function was considered the primary outcome of the programme. Patients were assessed for their sitting, standing and walking tolerance. Oswestry Disability Index (ODI) and Spinal Function Sort Score (SFSS) were used to assess patient perceived disability.

**Results:**

One hundred and fifty-eight patients were recruited. After the programme, statistically significant improvement was found in ODI (47.5 to 45.0, *p* = 0.01) and SFSS (98.0 to 109.5, *p* <  0.001). There was statistically significant improvement (*p* <  0.01) in sitting, standing, walking tolerance and straight leg raise tests. 47.4% of the patients were able to meet their work demand. Multivariate logistic regression model (R^2^ = 59.5%, χ^2^ (9) = 85.640, *p* <  0.001) demonstrated that lower initial job demand level and higher patient-perceived back function correlated with greater likelihood of returning to work.

**Conclusion:**

The results of this study may support the use of this multidisciplinary programme to improve patient function and return to work.

## Introduction

Low back pain (LBP) is the leading cause of disability worldwide, accounting for 50 million years of disability with significant healthcare costs [1, 2]. The situation is similar in Asian populations, where LBP is the second leading cause of disability with 7.7 million years of disability, incurring a significant economic burden with LBP-related absenteeism [3, 4]. Rehabilitation and reintegration of these patients into the working population, therefore, is paramount to alleviating the enormous medical and social costs of LBP.

Particularly amongst this population, up to one-third of patients with acute LBP progress into chronic LBP (pain for duration of at least 3 months), rendering them more prone to developing a persistent course of pain, typically resistant to conventional treatments such as physiotherapy and analgesics [5, 6]. In addition to the physiological component of pain, there is often associated psychological factors, for example anxiety and catastrophization, which can impede recovery to a significant degree [7]. Therefore, a multidisciplinary biopsychosocial model of rehabilitation is often required in order to ameliorate the physiological, psychological and social disabilities of chronic LBP [8].

Over the years, considerable effort has been placed into identifying suitable strategies to rehabilitate this group of patients with chronic LBP. In the recent literature, there is a general trend to migrate from single therapies such as physiotherapy, psychotherapy or medical treatment alone to multidisciplinary programmes in treatment of chronic LBP. A Cochrane review suggested that multidisciplinary rehabilitation is superior to usual care in terms of symptom relief and functional recovery [9]. However, there is no clear evidence that multidisciplinary rehabilitation is superior in helping patients return to work (RTW) [10]. It is worth noting that one of the limitations in this recent Cochrane review identified most of the literature included in recent reviews to be of low to moderate quality due to heterogeneous patient populations and intervention protocols [11, 12]. Also, most of the literature is based on Caucasian populations.

The current study aims to report the southern Chinese experience with a 14-week intensive multidisciplinary programme aiming at functional recovery in patients with chronic LBP, and to determine the predictive factors for successful RTW.

## Methods

### Aims of this study

This was a retrospective study of a group of subjects with chronic LBP, who were enrolled in a 14-week rehabilitation programme specifically catered for treatment of chronic LBP. Subjects were enrolled consecutively into the study over the period of 1996–2014. The primary goal of the study was to report the functional improvement after the programme in terms of improvement in perceived disability. Secondary parameters such as pain, physical tolerance and psychological stresses were also reported. The secondary goal was to determine the ability of subjects to RTW. Secondary analysis was performed to identify factors affecting the ability to work in this particular group of subjects.

### Programme details

Subjects selected were enrolled in a 14-week rehabilitation programme in a tertiary centre. The aim of the programme was to restore function to these subjects so they could RTW. The programme was structured into integrated sessions which encompassed input from orthopaedic surgeons, physiotherapists, occupational therapists and clinical psychologists. Subjects attended whole-day outpatient sessions on a 3 times weekly basis in a government-funded rehabilitation facility for the programme. Subjects on attendance paid the usual government-subsidized fees for specialist clinic attendance. There were no other sources of private funding in the programme.

### Patient selection

Stringent inclusion and exclusion criteria were adopted in subject recruitment. Subjects with chronic non-specific LBP with failure of RTW were selected for admission into the programme. Chronic LBP was defined as LBP with duration of more than 3 months. Subjects recruited had been unable to return to duty due to chronic LBP for at least 3 months. All subjects had gone through a period of at least 3 months of conventional back physiotherapy or pharmacological treatment with residual LBP affecting RTW.

Subjects with neurological deficits from history or physical examination were excluded. Those who had congenital or structural spinal anomalies, history of previous spinal surgery or LBP related injections were excluded. Subjects who were unable to comply with regular attendance to therapy and assessment were excluded. Subjects showing signs of malingering were also excluded from the programme in study after thorough psychological screening.

Subject screening was performed in the specialist orthopaedic outpatient clinic. According to the aforementioned criteria and assessment by an experienced orthopaedic surgeon, subjects were referred to the programme and enrolled into study. At admission into the programme, the subjects were reviewed again by orthopaedic surgeons, physiotherapists, occupational therapists and clinical psychologists according to the inclusion and exclusion criteria.

### Assessment and outcome measures

Assessment time-points were fixed at baseline upon recruitment at the commencement of the rehabilitation programme and at 8-weeks and 14-weeks into the programme. Objective assessment of the subjects was performed by physiotherapists and occupational therapists, while patient-reported outcomes were gathered with questionnaires and patient-centred interviews with clinical psychologists.

Functional outcomes from the rehabilitation programme were examined in four major areas: physical assessment, pain severity, psychological adaptation and disability perception.

#### Physical assessment

Sitting, standing and walking tolerance were assessed, and straight leg raise (SLR) tests were performed for both legs at the set time-points. The measurements were performed by the same physiotherapists following up the subject. Premorbid work strength, derived from the subject’s premorbid duty was measured with the Physical Demand Characteristics of work (PDC) by occupational therapists [13, 14]. PDC was graded into ‘0 = sedentary’, ‘1 = light’, ‘2 = medium’, ‘3 = heavy’ and ‘4 = very heavy’ depending on the amount and frequency of physical labour (lifting, pushing and pulling tasks) involved in the described duty. The subject’s work strength was determined by PDC in accordance with premorbid duty specific tasks performed during occupational therapist assessment [15].

#### Pain severity

Pain severity was measured using the visual analogue score (VAS). We assessed the pain perception for the worst pain experienced by the subject at rest and under exertion (standard walking task for 100 m), on a continuous scale from 0 to 10, with 0 indicating no pain, and 10 indicating the worst pain experienced by the subject.

#### Psychological adaptation

For the psychological aspect, stress response and coping response to chronic LBP was measured using Acceptance of Illness Scale (AIS) [16, 17]. AIS is a questionnaire designed to measure disease acceptance in adult individuals. Using a standard 8-statement questionnaire, each given a grade from 1 to 5, the total score of AIS is between 8 to 40. A low AIS score shows lack of adjustment to disease, with no acceptance of the condition, whereas a high score indicates acceptance and adjustment towards disease. Subjects’ overall psychological wellbeing was assessed with the Bradburn Affect Balance Scale (BABS) [18–20]. BABS is a 10-statement questionnaire, where the subject either agrees to or disagrees with each statement. Each statement within the questionnaire carries equal weighing, with 5 statements carrying positive points and 5 carrying negative points. The total score of the BABS therefore ranges from − 5 to + 5, where a more positive score indicates a more satisfied and elated emotional state. Depressive tendencies were detected using the Beck Depression Inventory (BDI) [21, 22], a 21-question tool with each question given a grade from 0 to 3, to give a total score out of 63. The score placed the subject into groups of minimal, mild, moderate to severe depression. A higher score points to more depressive tendency within a subject. These parameters were gathered with use of questionnaires and interviews with clinical psychologists during each assessment.

#### Disability perception

Oswestry Disability Index (ODI) and Spinal Function Sort Score (SFSS) were used to gauge the subject’s subjective perception of disability. ODI is an index derived from the Oswestry Low Back Pain Questionnaire which is used to quantify disability of LBP [23]. The questionnaire contains ten items pertaining to areas of daily life concerning intensity of pain, lifting, sexual function, social life, sleep quality and ability to care for oneself, walk, sit, stand or travel. Each item is graded from 0 to 5 indicating least to severe degree of disability in the area. The score is summed and multiplied by two, with an index of 0 to 100, with 0 indicating no disability and 100 indicating maximal disability. SFSS is an assessment of perceived ability to perform work tasks involving the spine [24]. It consists of 50 graphically depicted tasks in which the subject is instructed to grade the ability to perform the task on a scale of 0 to 4, with 0 indicating inability and 4 indicating capability in performing the task. The total score of the SFSS ranges from 0 to 200, with a higher score indicating a better perceived capability in terms of physical demand.

### Returning to work

The subject’s ability to RTW was assessed using work strength progression in the form of PDC compared to the described job demand according to the subject. The subject was asked to perform the tasks described in the initial job description, with the performance graded according to PDC. Subjects with a grade higher or equal to that of the assessed job demand grade and successfully re-integrated into the same workplace were defined as capable of RTW. This was assessed at the completion of the programme. In contrary, subjects with a performance lower than that of the described job demand were defined as failing to RTW.

### Statistical analysis

Descriptive statistics were calculated and presented in mean, standard deviation and percentage. Normality tests were conducted using Shapiro-Wilk tests and scatter plots. For the comparison of parameters before and after the programme, paired t-test and its non-parametric equivalent Wilcoxon signed rank test were conducted where appropriate. Correlation tests of the outcome of RTW with various parameters was conducted using chi-square test of independence (for categorical variables) and Spearman’s rank-order correlation (for continuous variables). For the prediction of whether patients have the ability to RTW after the programme, the ability to return to work was dichotomized as capable (coded as 1) and failure (coded as 0). Univariate analysis was run using logistic regression between each factor with the outcome defined. The pre-selection significance level was set at 0.20 for factors to be included in the multivariate regression models [25]. Multivariate logistic regression was then performed to ascertain the effect of various factors on the likelihood of subjects returning to work. Receiver operating characteristic (ROC) curve was utilized to evaluate the multivariate logistic regression model. Sensitivity and specificity in predicting the possibility of RTW post-programme was reported. Odds ratios (ORs) and 95% confidence intervals (CIs) were reported as appropriate. Statistical significance was considered with a *p*-value < 0.05. Statistical analyses were conducted using SPSS Windows 24.0 (IBM SPSS Inc., Chicago, Illinois).

## Results

**Fig. 1:**
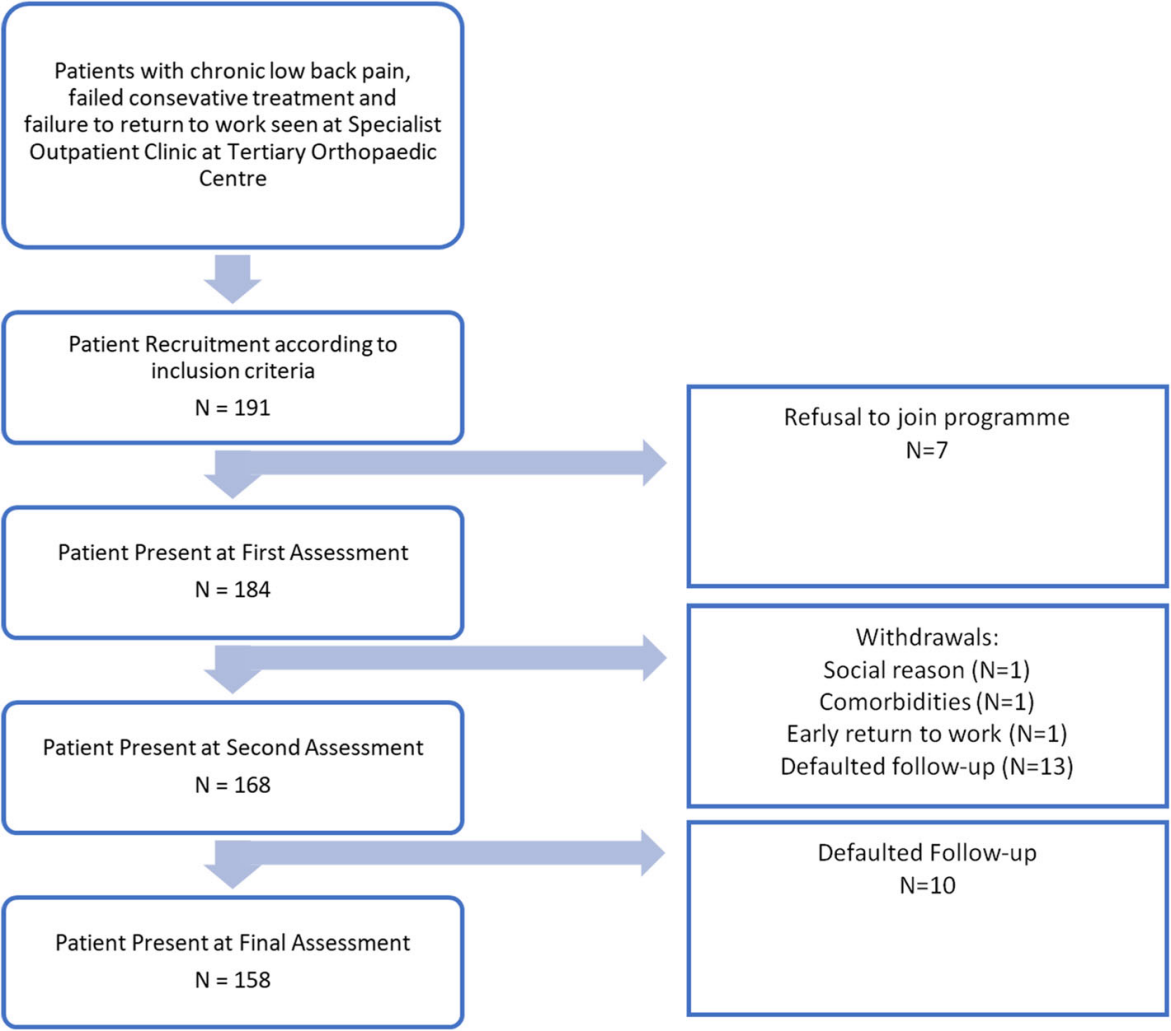
The final available number of subjects for analysis was 158 after multiple defaulters and withdrawals from the programme

One hundred and ninety-one subjects were enrolled into the programme but only 158 subjects were accrued for final analysis (Fig. 1). Mean age was 41 ± 9.4 years old, with 41 female subjects (25.9%) and 117 male subjects (74.1%). IOD was reported in 144 subjects (91.1%) as the cause for their chronic LBP. One hundred and fifty-three subjects (96.8%) have received a combination of pharmacological treatment and physiotherapy prior to admission into the programme, while the remaining 5 subjects (3.2%) received only analgesics without attendance to physiotherapy. Majority of our subjects had a medium to heavy job demand according to PDC.

### Functional assessment outcomes (Table 1)

#### Physical assessment

Sitting tolerance improved from 50.0 to 61.6 min, standing tolerance improved from 40.7 to 47.2 min, while walking tolerance improved from 50.6 to 59.7 min (*p* <  0.001) after the multidisciplinary rehabilitation programme. SLR results improved from 74 to 77 degrees on both sides (*p* <  0.05). The median work strength improved from 1 to 2 (from ‘light’ to ‘medium’ grade) after the programme (*p* < 0.001).

**Table 1:** Change in Outcomes after the Multidisciplinary Programme

	Pre-programme	Post-programme	*p*-value
*Physical Assessment*			
Sitting Tolerance (min)	50.0 ± 43.2	61.6 ± 47.3	0.001*
Standing Tolerance (min)	40.7 ± 41.2	47.2 ± 41.8	0.001*
Walking Tolerance (min)	50.6 ± 47.3	59.7 ± 48.8	< 0.001*
SLR – Right (deg)	74 ± 13.3	77 ± 14.0	0.008*
SLR – Left (deg)	74 ± 14.1	77 ± 14.0	0.001*
Work Strength	1 (‘Light’)	2 (‘Heavy’)	< 0.001*
*Pain Severity*			
VAS at rest	3.8 ± 2.3	3.8 ± 2.3	0.754
VAS on exertion	6.8 ± 2.1	6.8 ± 2.0	0.862
*Psychological Adaptation*			
AIS	21.4 ± 6.2	22.6 ± 7.0	0.002*
BABS	−2.1 ± 1.9	− 2.0 ± 2.2	0.886
BDI	17.2 ± 9.8	19.6 ± 11.8	0.002*
*Disability*			
ODI	47.5 ± 13.1	44.0 ± 16.4	0.013*
SFSS	97.9 ± 39.6	109 ± 42.5	< 0.001*

*SLR* Straight leg raising test, *VAS* Visual analogue scale, *AIS* Acceptance of Illness Scale, *BABS* Bradburn Affect Balance Scale, *BDI* Beck Depression Inventory, *ODI* Oswestry Disability Index, *SFSS* Spinal Function Sort Score

* denotes *p* < 0.05

#### Pain severity

After completion of the multidisciplinary programme, there was no significant change in VAS at rest (*p* = 0.75) and under exertion (*p* = 0.86). Their values have remained similar pre-programme and post-programme.

#### Psychological adaptation

Subjects generally showed improved acceptance towards their chronic LBP as shown by the improved AIS from 21 to 22 (*p* < 0.05). There was deterioration in BDI from 17 to 19 (*p* < 0.05). The general mood and emotional wellbeing as represented by BABS showed no significant change before and after the programme (*p* = 0.89).

#### Disability perception

There was improvement in subjects’ perception on their own disabilities in work and activities of daily living. This was reflected by the improvement in the ODI from 47.5 to 44.0% (*p* < 0.05) and SFSS scores from 97.9 to 109 (*p* < 0.001).

### Return to work and factors determining likelihood of return to work

**Fig. 2:**
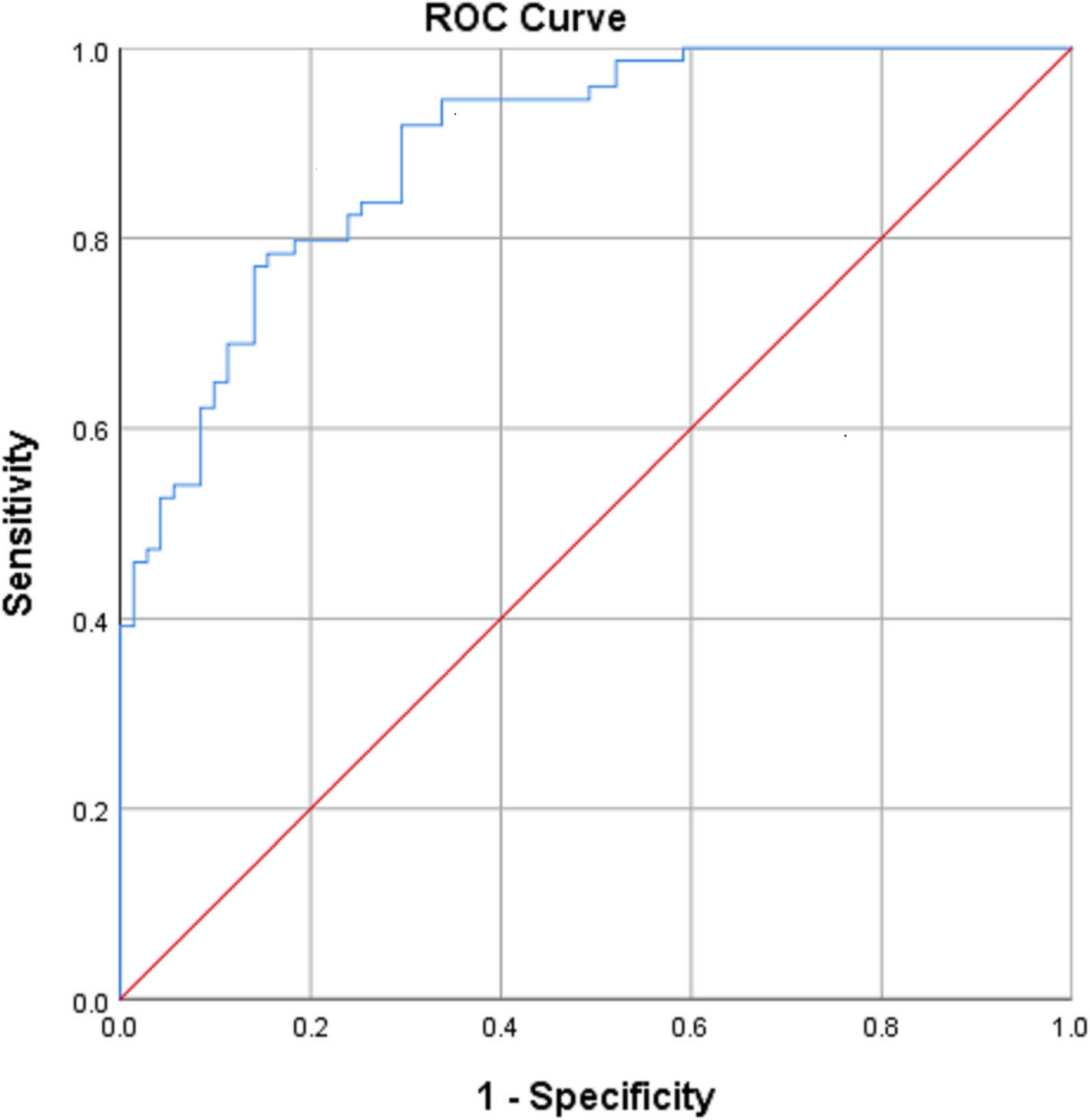
ROC curve showed 82.4% sensitivity and 76.1% specificity for predicting the possibility of returning to work post-programme with a cut-off value of 0.51 predicted probability generated for the multivariate logistic regression model

After the programme, 48.1% of subjects met their work demand. Univariate analyses (Table 2) showed that age, sex, initial job demand level, smoking, IOD status, pre-programme work strength, pre-programme BDI and pre-programme BABS were correlated with ability to RTW. Univariate analysis (Table 3) revealed that job demand level, smoking, IOD, pre-programme BDI, SFSS and BABS were significant factors (*p* < 0.20). Multivariate regression defined a statistically significant model, χ^2^ (9) = 85.640, *p* < 0.001. The model explained 59.5% (Nagelkerke R^2^) of the variance and correctly classified 78.6% of cases (Table 4). Statistically significant effect on ability to RTW was found with initial job demand level (by PDC) at medium and heavy levels, and higher pre-programme SFSS. The initial job demand (by PDC) was found to be the most significant predictor for whether the patients RTW. Initial job demand of medium and heavy levels has the greater likelihood of returning to work in this study cohort in comparison to those with very heavy job demand. However, it was observed that the upper and lower bounds of the 95% CI of the OR covered a wide range, indicating a less precise estimate of the OR. The pre-programme SFSS also associated with RTW, with every 1 score increase in SFSS leading to 1.3% increase in the odds of RTW (OR = 1.013, 95% CI 1.001–1.026, *p* = 0.037). Smoking status, pre-programme BABS, BDI as well as the status of whether the cause of LBP was an IOD did not show any significant effect on the ability to RTW. ROC analysis (Fig. 2) showed 82.4% sensitivity and 76.1% specificity for predicting the possibility of returning to work post-programme with a cut-off value of 0.51 predicted probability generated for the multivariate logistic regression model.

**Table 2:** Correlation of various parameters with ability to return to work

Parameters	Coefficient^a^	*p*-value
*Categorical Parameters*		
Gender	−0.102	0.199
Job demand level	−0.604	< 0.001
Smoking	−0.121	0.127
Education	0.019	0.820
IOD	−0.150	0.060
*Continuous Parameters*		
Age	0.098	0.200
Pre-programme sitting tolerance	−0.069	0.389
Pre-programme standing tolerance	−0.064	0.426
Pre-programme walking tolerance	−0.059	0.459
Pre-programme VAS at rest	−0.050	0.534
Pre-programme VAS on exertion	−0.100	0.213
Pre-programme ODI	0.014	0.610
Pre-programme BDI	0.018	0.104
Pre-programme SFSS	0.004	0.118
Pre-programme BABS	0.091	0.071

*IOD* Injury on duty, *VAS* Visual analogue scale, *ODI* Oswestry Disability Index, *BDI* Beck Depression Inventory, *SFSS* Spinal Function Sort Score, *BABS* Bradburn Affect Balance Scale

^a^ Coefficient from Chi-square test of independence/Fisher’s exact test and Spearman’s rank-order correlation depending on type of parameters

**Table 3:** Univariate analysis of various parameters with ability to return to work

Parameters	Standard error	OR (95%CI)	*p*-value
Age	0.017	1.018 (0.984–1.052)	0.304
Gender (ref. females)	0.365	1.543 (0.754–3.158)	0.235
Job demand level	- (multi-levels)		0.001
Smoking (ref. smoker)	0.323	0.648 (0.344–1.221)	0.179
Education	- (multi-levels)		0.963
IOD (ref. Yes)	0.615	2.955 (0.885–9.858)	0.078
Pre-programme work strength	40,192.962	161 × 10^7^	1.000
Pre-programme sitting tolerance	0.004	1.000 (0.992–1.007)	0.933
Pre-programme standing tolerance	0.004	0.998 (0.991–1.006)	0.697
Pre-programme walking tolerance	0.003	1.000 (0.993–1.000)	0.891
Pre-programme VAS at rest	0.070	0.962 (0.838–1.104)	0.578
Pre-programme VAS on exertion	0.077	0.943 (0.811–1.095)	0.442
Pre-programme ODI	0.012	0.991 (0.968–1.015)	0.476
Pre-programme BDI	3.280	0.970 (0.938–1.003)	0.070
Pre-programme SFSS	0.004	1.010 (1.001–1.018)	0.022
Pre-programme BABS	0.085	1.130 (0.957–1.130)	0.150

*IOD* Injury on duty, *VAS* Visual analogue scale, *ODI* Oswestry Disability Index, *BDI* Beck Depression Inventory, *SFSS* Spinal Function Sort Score, *BABS* Bradburn Affect Balance Scale

**Table 4:** Multivariate analysis of ability to return to work

Parameters	Coefficient	Standard error	Wald	*p*-value	Odds Ratio	95% CI
Initial Job Demand (ref.: very heavy)			20.779	< 0.001*		
Sedentary	24.035	27,721.7	0.000	0.999	2.742 × 10^10^	
Light	24.806	8502.8	0.000	0.998	5.929 × 10^10^	
Medium	4.011	0.880	20.763	< 0.001*	55.199	9.833–309.880
Heavy	3.075	0.815	14.242	< 0.001*	21.658	4.385–106.969
Smoking (ref.: Smoker)	0.224	0.500	0.201	0.654	1.251	
IOD (ref.: Yes)	0.260	0.943	0.076	0.783	1.296	
Pre-programme BDI	−0.022	0.031	0.495	0.482	0.979	
Pre-programme SFSS	0.013	0.006	4.355	0.037*	1.013	1.001–1.026
Pre-programme BABS	0.214	0.153	1.963	0.161	1.239	

*SFSS* Spinal Function Sort Score, *BDI* Beck Depression Inventory, *BABS* Bradburn Affect Balance Scale

* denotes *p*-value < 0.05

## Discussion

Chronic non-specific LBP is a significant disease entity with heavy social, economic and medical costs and implications. Current evidence suggests that a multi-disciplinary model is helpful in achieving reduction in pain and symptoms, however there is a lack of understanding of predictive factors of outcomes related to RTW [26]. The first part of this study provides insight on the effectiveness of a multidisciplinary programme for this group of subjects, with a particular aim for functional recovery. From the above data, there was functional improvement in this group of subjects with chronic non-specific LBP. In the second part of the study, we have demonstrated that subjects with a lower initial job demand level and higher baseline back-specific function were more likely to be able to RTW. This result aids identification of individuals with good potential and higher likelihood of success and may benefit from an earlier and more aggressive form of multidisciplinary training.

The data gathered from this study shows that the multidisciplinary programme improved physical tolerances towards daily activities in patients with chronic non-specific LBP. This correlates with overseas and local studies, where similar improvements in physical outcomes were observed after multidisciplinary rehabilitation [9, 10, 27]. There was also a significant improvement in work tolerance from ‘light’ grade to ‘medium’ grade duty according to PDC classification. Physical improvements aside, improvement of the perceived function in daily living corresponded to that in previous studies with better ODI and SFSS [9, 27]. Although the change in ODI did not reach the minimum clinically important difference (MCID) of 17 [28], some studies suggested that ODI alone does not provide a comprehensive assessment of disability [29, 30]. Therefore, with the inclusion of SFSS to assess back and spine specific function, our study hopes to more accurately reflect the functional capability of patients. However, in the psychological aspect, despite improvements in physical and self-perceived capabilities, increase in reported BDI suggested pervasive negative cognitions and ideas, with a mean BDI within the mild depression category at pre-programme and post-programme assessment. This change however was not clinically significant according to the proposed MCID of 17% change as discussed in the recent literature [31]. Moreover, they were more accepting towards chronic LBP as evident by the AIS scores reported.

The generated statistical model in this study identified the patient with a lower initial job demand and a better self-perceived back-specific functional capacity (as evident by SFSS) to be more likely to achieve RTW. The initial job demand level was found to have a statistically significant correlation with the ability to work. Subgroup regression analysis based on job demand level by PDC classification further determined that patients with ‘medium’ grade of initial job demand are most likely to RTW. This also agrees with overseas studies suggesting that a lower job physical demand tends to increase RTW [2, 32, 33]. A better patient-perceived back-specific functional capacity was also noted to have a significant correlation to the ability to RTW, which was supported by current literature [2, 32, 34]. Surprisingly, the status of IOD and its implied associated compensation did not influence the likelihood to RTW as compared to some studies [32, 35]. This can potentially provide a different perspective as to the need to differentiate this group of patients when treating chronic LBP, as suggested by previous studies [2, 27]. The factors identified in the results may provide a guide to future resource allocation focusing on functional rehabilitation in patients with chronic LBP.

### Limitations

There are some limitations to the study which could be improved. Firstly, the study was retrospective in design, which potentially introduced selection bias into the study. The preset inclusion and exclusion criteria might select a group of subjects who might have social and psychological confounders that could hinder accurate assessment of their rehabilitation progress, as well as their ability to RTW. Secondly, the study was carried over a long period of time of over 18 years, which could affect the consistency and fairness of assessment for all subjects. Despite the same set of assessment tools and rehabilitation protocols, personnel and facility changes could still bring in variability to the programme delivered. Thirdly, our study was of a cross-sectional study design, which means that there was no control group to compare the outcome of RTW and improvement after rehabilitation programme. The study could only report the observed RTW rate which was then used to identify common factors in those who were able to RTW. The analysis is further limited due to only 158 assessed at follow-up.

### Further directions

There are a few areas that need to be further investigated prior to bridging the model generated within this study to predict RTW. Psychological assessment tools in this study were mainly for assessment of general emotional wellbeing as well as acceptance towards general diseases. In spite of this, they may not completely represent the complete psychological response to pain for analysis, in particular the fear-avoidance tendencies in patients with chronic pain [36]. Further studies may consider adaptation of more pain specific questionnaires, for example Fear-Avoidance Beliefs Questionnaire (FABQ) to ascertain the psychological component of chronic non-specific LBP [37, 38]. Also, from previous studies stratifying the probability of returning to work in this particular population of patients with chronic LBP, social circumstances and psychological expectations on RTW were found to correlate with participation in patients with chronic non-specific LBP, which have not been covered in this study [2, 39]. According to a recent review on factors obstructing return to functional roles in patients with LBP, workplace factors, job satisfaction and employer-employee communications were also suggested to be underlying factors affecting RTW [40]. Moreover, with a focus on social reintegration, more longitudinal follow-up may be needed to delineate the time in which the patients achieve RTW and subsequent maintenance, which accounts for significant years of disability and social costs. Last but not least the cost and benefit analysis of this multidisciplinary mode of rehabilitation may be another factor to take into account when adopting this model in rehabilitating patients with chronic LBP.

## Conclusion

We report from this study the outcomes of the multidisciplinary programme, which showed improvement in function and general wellbeing of a patient group that had failed other forms of conservative treatment and was unable to RTW. We have identified several predictors of good outcomes for successful functional rehabilitation and matching workplace demands, namely a low initial job demand, and a higher patient-perceived back function. A viable model was constructed with our data to identify patients with good potential most suitable for this intensive rehabilitation programme. Further investigation may be beneficial into psychological and social elements affecting the likelihood of returning to work in this group of patients.

## Data Availability

The datasets used and/or analysed during the current study are available from the corresponding author on reasonable request.

## Abbreviations

AIS: Acceptance of Illness Scale
BABS: Bradburn Affect Balance Scale
BDI: Beck Depression Inventory
CI: Confidence interval
FABQ: Fear-Avoidance Beliefs Questionnaire
IOD: Injury on duty
LBP: Low back pain
MCID: Minimum clinically important difference
ODI: Oswestry Disability Index
OR: Odds ratio
PDC: Physical demand characteristics of work
RTW: Return to work
SFSS: Spinal Function Sort Score
SLR: Straight leg raise test
VAS: Visual analogue scale
